# Vibration Fatigue of FDM 3D Printed Structures: The Use of Frequency Domain Approach

**DOI:** 10.3390/ma15030854

**Published:** 2022-01-23

**Authors:** Massimiliano Palmieri, Guido Zucca, Giulia Morettini, Luca Landi, Filippo Cianetti

**Affiliations:** 1 Deparment of Engineering, University of Perugia, Via G. Duranti 93, 06125 Perugia, Italy; giulia.morettini@unipg.it (G.M.); luca.landi@unipg.it (L.L.); filippo.cianetti@unipg.it (F.C.); 2 Italian Air Force, Aeronautical and Space Test Division, Via Pratica di Mare, 00040 Pomezia, Italy; guido.zucca@aeronautica.difesa.it

**Keywords:** vibration fatigue, 3D printed structures, fatigue damage, spectral methods

## Abstract

Additive manufactured structures are replacing the corresponding ones realized with classical manufacturing technique. As for metallic structures, 3D printed components are generally subjected to dynamic loading conditions which can lead to fatigue failure. In this context, it is useful, and sometimes mandatory, to determine the fatigue life of such components through numerical simulation. The methods currently available in literature for the estimation of fatigue life were originally developed for metallic structures and, therefore, it is now necessary to verify their applicability also for components fabricated with different materials. To this end, in the current activity three of the most used spectral methods for the estimation of fatigue life were used to determine the fatigue life of a 3D printed Y-shaped specimen realized in polylactic acid subjected to random loads with the aim of determining their adaptability also for this kind of materials. To certify the accuracy of the numerical prediction, a set of experimental tests were conducted in order to obtain the real fatigue life of the component and to compare the experimental results with those numerically obtained. The obtained outcomes showed there is an excellent match between the numerical and the experimental data, thus certifying the possibility of using the investigated spectral methods to predict the fatigue life of additive manufactured components.

## 1. Introduction

Despite additive manufacturing technology was limited to prototyping, in recent years the engineering panorama is witnessing to a growing application of this fabrication technique in various sectors. It is quite common, indeed, the use of 3D printed products in the biomedical sector [1,2], for the production of prostheses, in the automotive sector for manufacturing parts such as engine blocks or frame [3,4] or in the aerospace sector [5,6]. The application of additive manufacturing in various sectors mainly depends on the intrinsic advantages of such technology. In fact, additive manufacturing offers the possibility to create very complex geometry parts, sometimes not doable with conventional production techniques, or the possibility to print smart materials thus creating smart structures, 3D printed sensors and MEMS [7,8,9,10,11,12,13,14,15,16,17,18,19,20]. In addition to such advantages in terms of geometry freedom and application, the constant growing of this technology is also due to the production costs that are considerably reduced and the dwindled manufacturing wastes [21].

Thanks to the considerable benefits offered by the additive manufacturing technology, it is now common practice to replace components realized with conventional fabrication technique with the 3D-printed counterparts. Due to the growing diffusion of this technology, both regarding metal alloys and thermoplastic materials, it is becoming necessary to standardize, as done for conventional manufacturing techniques, the production processes, materials and structural tests especially for all those applications considered critical for human safety. In this context, engineers and researchers are focusing their research activities on the control of the printing process since it highly affects the structural strength of the final printed component. In this context, most of the available researches are voted to exploit the image analysis, machine learning and neural network to monitor the printing process in order to reduce, in such a way, the probability of an anomaly within the printed structure [22,23,24,25].

Beside previous aspects, the mechanical characterization of the materials used in 3D printing and the study of the influence of the printing parameters on the structural strength, both static and dynamic, is of considerable interest [26,27,28,29,30].

Due to the constant increasing use of 3D printed components in real application, additive manufactured parts are often subjected to strong dynamic loading conditions, such as vibrations, that in many cases may result to be particularly burdensome [31]. For this reason, any mechanical component is subjected to vibration tests aimed to evaluate its structural strength. As for classically manufactured components, in order to reduce production and testing times and costs, numerical simulation may be a useful tool also for 3D printed products [32,33]. By simulating whichever test conditions, it is possible to iterate any design choice without the need of prototyping and testing, hence saving times and costs. The prototyping and qualification test phases may therefore be executed only when an optimal design is achieved reducing, in such a way, the risk of unexpected failure during the qualification tests imposed by current standard references. However, to be the numerical analysis a benefit in the design process, it must be reliable in terms of results and computationally efficient. The frequency domain approaches for structural analysis has intrinsically both of these properties [34,35,36].

Despite the intensive used of spectral methods in vibration fatigue for metal alloy components, literature is poor of their application for PLA 3D printed components. For this reason, the present activity investigates the possibility of using classical spectral methods, although initially developed for metal alloys, to estimate the fatigue life of components manufactured in polylactic acid (PLA) material subjected to random loads. To this aim, a finite elements model of a Y-shaped specimen was created and the fatigue life was estimated in frequency domain with three of the most used methods: the narrow-band method, the Tovo-Benasciutti method and the Dirlik method [37]. The numerical analysis were carried out by exciting the specimen with random loads, defined by three different RMS level but characterized by the same bandwidth, properly drawn to break the component in a reasonable time. To validate the ability of the adopted spectral methods to correctly predict the fatigue life of the components, experimental tests were carried out to measure the actual fatigue life of a set of 3D printed PLA samples. The obtained results indicate there is a good accuracy of the spectral methods used in this activity to predict the fatigue life also of PLA 3D printed structures for all the considered excitation profile. The maximum percentage error committed by the methods is always lower than %, thus certifying a good accuracy of the investigated approaches.

This manuscript is organized as follow: Section 2 introduced theoretical aspects of signal processing, structural dynamics and fatigue calculation in frequency domain; Section 3 introduced the numerical activity and the obtained results while Section 4 described the experimental tests and shows comparisons between numerical and experimental results. Section 5 draws the conclusion.

## 2. Theoretical Background

In this section, theoretical concepts of signal analysis, structural dynamics and the main methods for the fatigue damage calculation in frequency domain are illustrated.

### 2.1. Properties of Random Processes

A random process x(t) is fully represented in frequency domain by the Power Spectral Density (PSD) $S_{x}\left(f\right)$ where *f* denotes the frequency vector. In fatigue application, the one-side PSD, thus defined only on the positive half-axis, is generally used. A PSD is further completely described by the spectral moments defined in Equation (1) [38].

(1) $$m_{i}=\int_{0}^{\infty }f^{i}S_{x}\left(f\right)df$$

The assessment of spectral moments $m_{i}$ allows defining the statistical properties of a random process. For example, for a zero-mean stationary Gaussian process, the zero-order spectral moment $m_{0}$ equals the root mean square (RMS) of the process and thus the variance.

From the spectral moments, it is also possible to calculate the number of zero crossing with a positive slope per unit of time $\nu_{0}$ and the number of maxima per unit of time $\nu_{p}$:

(2) $$\nu_{0}={\left(\frac{m_{2}}{m_{0}}\right)}^{1/2}\;\nu_{p}={\left(\frac{m_{4}}{m_{2}}\right)}^{1/2}$$

In vibration fatigue, the irregularity factor γ and the bandwidth parameter ε are generally used to characterize a random process. These parameters can be computed by the spectral moments as follows:

(3) $$\gamma =\frac{m_{2}}{{\left(m_{0}m_{4}\right)}^{1/2}}\;\varepsilon ={\left(1-\gamma^{2}\right)}^{1/2}$$

The bandwidth parameter ε allows to describe a process as to be narrow or wide band. It assumes values between 0 and 1, it is close to 1 for a narrow-band process and decreases as the bandwidth of the process increases [38,39]. Beside describing a random process, these parameters are fundamental for the choice of the appropriate spectral method to use for a correct fatigue damage estimation. However, the foundation of all spectral methods is the definition of the probability density function of the peaks p(x). For a generic random process, the probability density function has been introduced by Rice [40,41] and it takes the following form:

(4) $$p\left(x\right)=\frac{\sqrt{1-\gamma^{2}}}{\sqrt{2\pi m_{0}}}e^{-\frac{x^{2}}{2\left(1-\gamma^{2}\right)m_{0}}}+\gamma \frac{x}{m_{0}}e^{-\frac{x^{2}}{2m_{0}}}\Phi \left(\frac{\gamma x}{\sqrt{\left(1-\gamma^{2}\right)m_{0}}}\right)$$

where Φ is the standard normal distribution function. It is easy to note that for a narrow-band process, the probability density function shown in Equation (4) tends to the Rayleigh distribution, while for a wide-band process p(x) tends to the Gaussian distribution.

### 2.2. Structural Dynamics

In real applications, structural components generally show several degree of freedom and thus, the associated equation of motion can be deduced by the 2nd Newton’s law [42]:

(5) $$\left[M\right]\left\{\ddot{x}\right\}+\left[C\right]\left\{\dot{x}\right\}+\left[K\right]\left\{x\right\}=\left\{F\right\}$$

Equation (5) represents a set of couple differential equations that can be solved separately by rewriting it in a modal space imposing $\left\{x\right\}=\left[\phi \right]\left\{q\right\}$; where $\left[\phi \right]$ is the matrix of modal shapes and {q} are the generalized coordinates. Equation (5) thus takes the form:

(6) $$\left[I\right]\left\{\ddot{q}\right\}+\left[2\xi \omega_{0}\right]\left\{\dot{q}\right\}+\left[\omega_{0}^{2}\right]\left\{q\right\}={\left[\phi \right]}^{T}\left\{F\right\}$$

where $\left[I\right]$ is the identity matrix, $\left[2\xi \omega_{0}\right]$ is the diagonal matrix of damping in which ξ is the percentage damping, $\left[\omega_{0}^{2}\right]$ is the diagonal matrix of eigenvalues and ${\left[\phi \right]}^{T}\left\{F\right\}$ is the generalized force vector. Each of the Equation (6) represents a single degree of freedom system and can be solved separately and then combined according to the superposition principle in order to obtain the physical response. However, a faster method is to further rewrite Equation (6) in a state space form [43] according to the following notation:

(7) $$\begin{array}{cc} \left\{\dot{z}\left(t\right)\right\} & =\left[A\right]\left\{z\left(t\right)\right\}+\left[B\right]\left\{f\left(t\right)\right\}\\ \left\{y(t)\right\} & =\left[C\right]\left\{z\left(t\right)\right\}+\left[D\right]\left\{f\left(t\right)\right\} \end{array}$$

where $\left\{\dot{z}\left(t\right)\right\}=\left\{\left\{\dot{q}\left(t\right)\right\},\left\{\ddot{q}\left(t\right)\right\}\right\}$ represents the matrix of the state of the system while {y(t)} represents the matrix of the output. For a multi-degree of freedom system subjected to a force vector, the matrix $\left[A\right]$ and $\left[B\right]$ assume the following form:

(8) $$\begin{array}{cccc} \left[A\right] & =\left[\begin{array}{cc} \left[0\right] & \left[I\right]\\ \left[\omega_{0}^{2}\right] & -\left[2\xi \omega_{0}^{2}\right] \end{array}\right] & \left[B\right] & =\left[\begin{array}{c} \left[0\right]\\ \left[\phi \right] \end{array}\right] \end{array}$$

while the matrix $\left[C\right]$ and $\left[D\right]$ must be defined according to the required output. For the evaluation of fatigue damage, it is necessary to compute the stress response. To this aim, the output of the system described in modal shapes must be the generalized coordinates {q(t)}. Thus, $\left[C\right]$ and $\left[D\right]$ take the following form:

(9) $$\left[C\right]=\left[\left[I\right];\left[0\right]\right]\;\left[D\right]=\left[0\right]$$

To solve the problem in frequency domain, the frequency response function matrix $\left[H_{q}\left(\omega \right)\right]$ between the generalized coordinates and the input force must be addressed. Once the state matrix are known, the matrix $\left[H_{q}\left(\omega \right)\right]$ is easily obtainable as:

(10) $$\left[H_{q}\left(\omega \right)\right]=\left[C\right]\cdot {\left(j\omega \left[I\right]-\left[A\right]\right)}^{-1}\left[B\right]$$

Once the frequency response function $\left[H_{q}\left(\omega \right)\right]$ is known, the Power Spectral Density of stress $\left[S_{\sigma }\left(\omega \right)\right]$ can be computed according to Equation (11).

(11) $$\left[S_{\sigma }\left(\omega \right)\right]=\left[\phi^{\sigma }\right]\cdot \left[\left[H_{q}\left(\omega \right)\right]\left[S_{F}\left(\omega \right)\right]{\left[H_{q}\left(\omega \right)\right]}^{T}\right]\cdot {\left[\phi^{\sigma }\right]}^{T}$$

In Equation (11), $\left[\phi^{\sigma }\right]$ is the matrix of stress modal shapes of a selected element derivable from a finite element model and $\left[S_{F}\left(\omega \right)\right]$ is the Power Spectral Density of the excitation force. In case the analysed set of elements are subjected to a multiaxial stress state, the matrix of Power Spectral Density $\left[S_{\sigma }\left(\omega \right)\right]$ must be reduced into an equivalent one. Although different methods are available in literature [44,45,46], the one proposed by Pitoiset may be used [47]. This method foresees to evaluate an equivalent uniaxial stress Power Spectral Density as follows:

(12) $$S_{\sigma }^{eqv}\left(\omega \right)=Trace\left[\left[Q\right]\cdot \left[S_{\sigma }\left(\omega \right)\right]\right]$$

where the matrix [*Q*] is the Von Mises definition in frequency domain.

Once the equivalent uniaxial stress Power Spectral Density is known, it is possible to evaluate the fatigue damage according to the method introduced in the next section.

### 2.3. Spectral Methods for Fatigue Damage Estimation

The estimation of the fatigue behavior of mechanical components subjected to random loads is based on the assumption of linearity of the systems and that the cycles, associated to process, are Gaussian distributed. All spectral methods directly link the distribution of the cycles to the Power Spectral Density of the signal as shown in Section 2.1. There are several spectral methods in literature [37] but among them, only three of the most adopted in vibration fatigue were considered: the Narrow-Band method (NB), the Tovo-Benasciutti method (TB) and the Dirlik method (DK). The first one identifies the distribution of the cycles as to be a Rayleigh distribution, the second one applies a correction to the Narrow-Band method while the last one exploits a combination of different distributions to determine the actual cycles distribution. All three methods then combine the obtained cycles distribution with the fatigue strength of the material represented by the S-N curve described in Equation (13) and with the Palmgren-Miner rule [48].

(13) $$C=s^{k}N$$

where *C* and *k* are the material parameters, *N* is the number of cycles-to-failure and *s* represents the cycles amplitude. The fatigue damage *D* per unit of time, associated to a random stress state of an element constituting the model, can be computed using the Palmgren-Miner rule as follows [49]:

(14) $$D=\nu_{p}C^{-1}\int_{0}^{\infty }s^{k}p\left(s\right)ds$$

where $\nu_{p}$ is the number of maxima per unit of time given by Equation (3) and p(s) is the cycle amplitude probability density function of the stress cycles. Most of the spectral method are based on the well-known narrow-band approximation; thus, substituting p(s) of Equation (14) with the Rayleigh probability distribution deriving by Equation (4), the damage intensity for a narrow band process $D^{NB}$ can be computed as follows [50,51]:

(15) $$D^{NB}=\nu_{0}C^{-1}{\left(2m_{0}\right)}^{k}\Gamma \left(1+\frac{k}{2}\right)$$

where Γ(·) is the Euler gamma function.

The estimation of fatigue damage by Equation (15), however, is valid however only for narrow-band processes. Since wide-band processes often occur in nature, the overcoming of narrow-band approximation follows two main strategies: the first one concerns the use of correction coefficient to correct the fatigue damage estimated trough Equation (15), while the second foresees to define ad-hoc probability density function [52].

Among the available correction coefficient spectral methods, one of the most used is the one proposed by Tovo-Benasciutti [53] whom derived an expression for finding the expected rainflow fatigue damage for Gaussian loading. The fatigue damage is thus obtained as follows:

(16) $$D^{TB}=\left(b+\left(1-b\right)\alpha_{2}^{k-1}\right)\cdot D^{NB}$$

where:

(17) $$\begin{array}{cc} \; & \alpha_{1}=\frac{m_{1}}{\sqrt{m_{0}m_{2}}} \end{array}$$

(18) $$\begin{array}{cc} \; & \alpha_{2}=\frac{m_{2}}{\sqrt{m_{0}m_{4}}} \end{array}$$

(19) $$\begin{array}{cc} \; & b=\frac{\left(\alpha_{1}-\alpha 2\right)\left[1.112\left(1+\alpha_{1}\alpha_{2}-\left(\alpha_{1}+\alpha_{2}\right)\right)e^{2.11\alpha_{2}}+\left(\alpha_{1}-\alpha_{2}\right)\right]}{{\left(\alpha_{2}-1\right)}^{2}} \end{array}$$

Probably the most famous empirical formula for approximating the rainflow amplitude distribution of biomdal process is the one proposed by Dirlik [54], which uses a combination of an exponential and two Rayleigh probability density functions:

(20) $$p\left(s\right)=\frac{1}{\sqrt{m_{0}}\left[\frac{G_{1}}{Q}e^{\frac{-Z}{Q}}\frac{G_{2}Z}{R^{2}}e^{\frac{-Z^{2}}{2R^{2}}}+G_{3}Ze^{\frac{-Z^{2}}{2}}\right]}$$

In Equation (20), *Z* is the normalized amplitude and $x_{m}$ is the mean frequency, as defined by the author of the method:

(21) $$Z=\frac{x}{\sqrt{m_{0}}}\;x_{m}=\frac{m_{1}}{m_{0}}{\left(\frac{m_{2}}{m_{4}}\right)}^{\frac{1}{2}}$$

The parameters to evaluate the Dirlik probability density function are:

(22) $$\begin{array}{ccc} \; & G_{1}=\frac{2\left(x_{m}-\alpha_{2}^{2}\right)}{1+\alpha_{2}^{2}}\; & G_{2}=\frac{\alpha_{2}-G_{1}+G_{1}^{2}}{1-R} \end{array}$$

(23) $$\begin{array}{ccc} \; & G3=1-G_{1}-G_{2}\; & R=\frac{\alpha_{2}-x_{m}-G_{1}^{2}}{1-\alpha_{2}-G_{1}+G_{1}^{2}} \end{array}$$

(24) $$\begin{array}{cc} \; & Q=\frac{1.25\left(\alpha_{2}-G_{3}-G_{2}R\right)}{G_{1}} \end{array}$$

Despite the possible combination between Equations (14) and (20), for the Dirlik method it is possible to derive a closed-form expression for the fatigue damage intensity:

(25) $$D^{DK}=C^{-1}\nu_{p}m_{0}^{\frac{k}{2}}\left[G_{1}Q^{k}\Gamma \left(1+k\right)+2^{\frac{k}{2}}\Gamma \left(1+\frac{k}{2}\right)\left(G_{2}R^{m}+G_{3}\right)\right]$$

In the next paragraph, the fatigue life of a laboratory structure is computed with Equation (15), (16) and (25) and the results are compared with those experimentally obtained.

## 3. Numerical Analysis

To evaluate the possibility of using spectral methods for fatigue damage calculation, a dynamic model of a simple structure was created and the results numerically obtained were subsequently compared with the experimental outcomes.

### 3.1. FE Model

**Figure 1:**
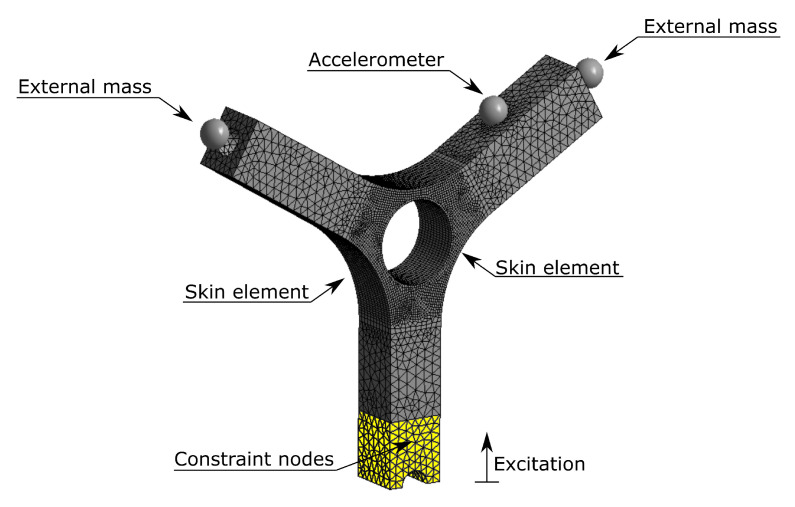
Finite element model of the Y-shaped specimen.

To verify the possibility of using spectral methods for fatigue life prediction, a dynamic model of a Y-shaped specimen, widely used in vibration fatigue, was considered (Figure 1) [55]. The finite element model foresees an elastic, homogeneous and isotropic linear material. For a proper dynamic modelling, the elastic modulus, the density and the percentage damping were experimentally obtained and the adopted procedure is detailed in next paragraphs. Nevertheless the used parameters are the following: elastic modulus equal to 2805 MPa, density equal to 1200 Kg·m^−3^ and percentage damping equal to 0.8%.

The model was meshed with solid elements with 10 nodes per element, on the external surfaces, around the central hole, skin elements (shell elements) were applied limiting the fatigue damage calculation was limited to those elements. The model is composed by 121,746 nodes and 43,652 elements.

To reduce the natural frequencies of the system, making them compatible with the available laboratory instrumentation, two masses were placed at 3.6 mm from the free ends of the specimen (Figure 1). The need of reducing the natural frequencies of the specimen by the use of external masses, is due to the mechanical properties of the PLA samples (in terms of mass and stiffness). Indeed the natural frequencies of the structure would assume high frequency values that would not be compatible with the available instrumentation. The masses were rigidly connected to the holes on the two arms of the specimen. A mass equal to 50 g was set for each external mass.

To better replicate such test conditions, an additional element mass, namely “Accelerometer” in Figure 1, has been applied to the point where the accelerometer will be placed in the experimental tests. The imposed mass of the accelerometer is supplied by the manufacturer and it is equal to 0.2 g. The mass is place at a distance equal to 1.4 mm in the normal direction to the surface corresponding to the position of the accelerometer center of gravity indicated in the datasheet.

To accurately reproduce the excitation conditions imposed by the electrodynamic shaker, the yellow part of the specimen shown in Figure 1 was constrained to the ground while the vertical translation was left free.

**Figure 2:**
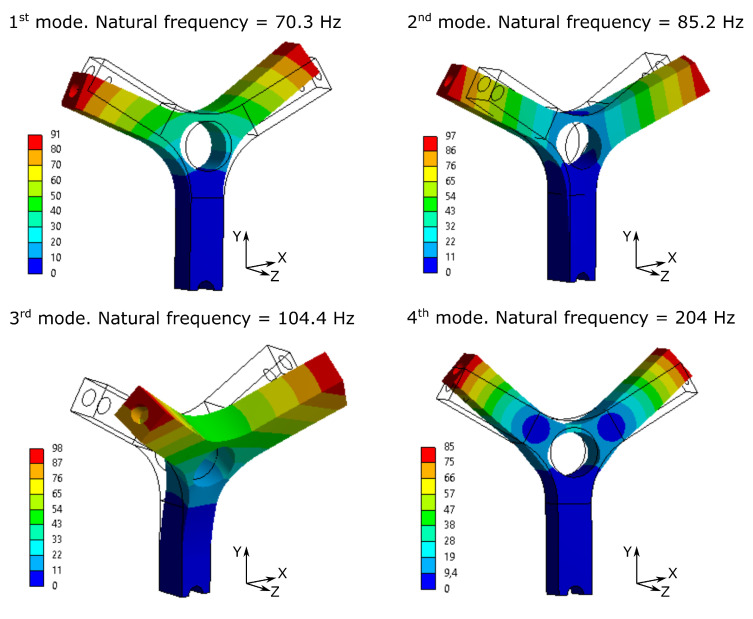
First four vibrating mode of the structure. Comparison between deformed and undeformed model.

A modal analysis was then performed in order to extract all the information needed to build a state space system, as illustrated in Section 2.2. From the FE model the first ten vibrating modes were extracted and all of the them were used in the dynamic analysis. Among them, Figure 2 shows the first four obtained vibrating modes, being them the most representative of the dynamic behavior of the structure.

Among the extracted vibrating modes, only the fourth one was chosen to be excited. The fourth vibrating mode is the symmetrical bending of the two arms as visible from Figure 2. To this end, an input Power Spectral Density (PSD) was designed, as detailed in next section, centered on the frequency corresponding to the fourth vibrating mode and equal to 204 Hz. With the designed excitation profile, only the fourt vibrating mode is excited and thus, the sample behaves like a single degree of freedom system, making the numerical and experimental results easily comparable.

### 3.2. Determination of Damping and Elastic Modulus through DMA Test

**Figure 3:**
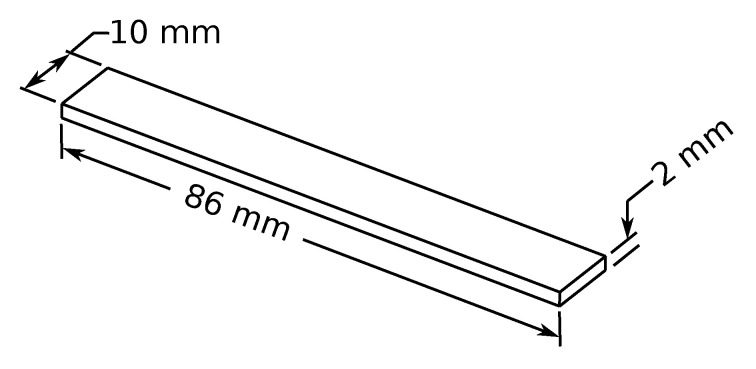
Specimen used for DMA test aimed to determine the elastic modulus and the damping ratio of the White Pearl PLA material.

To correctly represent the dynamic behavior of a mechanical structure, it is mandatory to assess the right value of the elastic modulus and the damping ratio [56]. To this aim, DMA (Dynamic Mechanical Analysis) was performed. The DMA specimens were produced in accordance with the geometric definition of ASTM D5023-15 [57] which defines the procedure to determine the mechanical properties of plastic materials within the region of linear viscoelastic behavior. The printed specimen is shown in Figure 3.

A set of three samples were horizontally printed (according to the printing parameter of the Y-shaped specimen detailed in Section 4.1). The excitation frequency was varied from 0 to 100 Hz and a constant room temperature was considered. The maximum amplitude displacement was limited to 500 um.

The DMA test permits the evaluation of the complex Elastic bending Modulus $E_{b}^{*}$ performing a 3-point bending test. This parameter can be defined as:

(26) $$E_{b}^{*}=E^{{}^{\prime }}+\iota E^{{}^{\prime \prime }}$$

where $E^{{}^{\prime }}$ and $E^{{}^{\prime \prime }}$ are the storage (also called elastic) modulus and the loss modulus, respectively. The storage modulus $E^{{}^{\prime }}$ describes the elastic property of the material while the loss modulus $E^{{}^{\prime \prime }}$ the viscoelastic properties. If the DMA tests are performed in the same direction as the bending test, in absence of other significant test results, the storage modulus $E^{{}^{\prime \prime }}$ is close to the bending elastic modulus $E_{b}$. This means the loss factor *i*, which defines the hysteresis of the viscoelastic processes, can be derived as:

(27) $$\iota =tan\left(\delta \right)=\frac{E^{{}^{\prime \prime }}}{E^{{}^{\prime }}}$$

where δ is the phase shift angle between the stress and strain.

As suggested by BS ISO 4664 standard [58], it is possible to correlate the loss factor ι with the ratio of decay of amplitude in the free vibration analysis Λ, defined as logarithmic decrements, which is a measure of energy dissipation in the time domain analysis [42]:

(28) $$\Lambda =\frac{\left(1-\sqrt{1-\iota^{2}}\right)2\pi }{\iota }$$

The logarithmic decrement Λ is dimensionless and is another form of the damping ratio ξ. Once Λ is known, ξ can be found by solving the Equation (6) [37]:

(29) $$\xi =\frac{\Lambda }{\sqrt{{\left(2\pi \right)}^{2}+\Lambda^{2}}}$$

**Figure 4:**
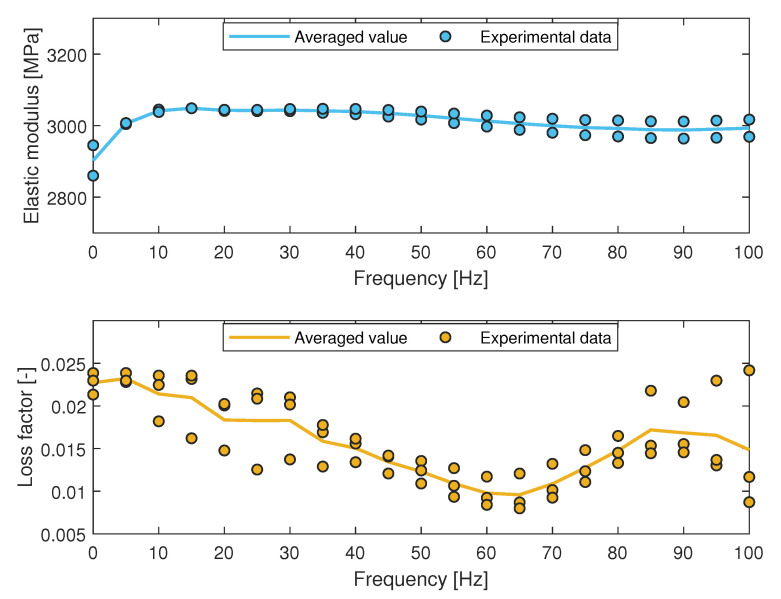
Elastic modulus and loss factor obtained from DMA.

The results in terms of the elastic modulus and the loss factor are shown in Figure 4.

In Figure 4, the points represent the experimental data, while the continuous line is the averaged value of experimental outcomes computed for each frequency. As visible in Figure 4, while the Elastic Modulus is outright constant over the frequency, the loss factor, and thus the damping ratio, shows unstable behavior in two main frequency ranges (within 20 Hz and from 60 Hz to 100 Hz). The unstable behavior of the loss factor in both highlighted frequency range can be related to the dynamics behavior of the specimens. In order to realize a FE model with homogeneous and linear-elastic behavior, it was necessary to obtain a single value for both the elastic modulus and the damping ratio. To this aim, the obtained outcomes were averaged over the considered frequency range [58,59] and the resulting values are shown in Table 1.

As shown in Table 1, the obtained averaged elastic modulus (equal to 2805 MPa) is in agreement with the value indicated by the manufacturer that corresponds to 2400 MPa. The gap between the obtained and supplied elastic modulus may be due to the different printing parameters.

### 3.3. Determination of the Material S-N Curve

**Figure 5:**
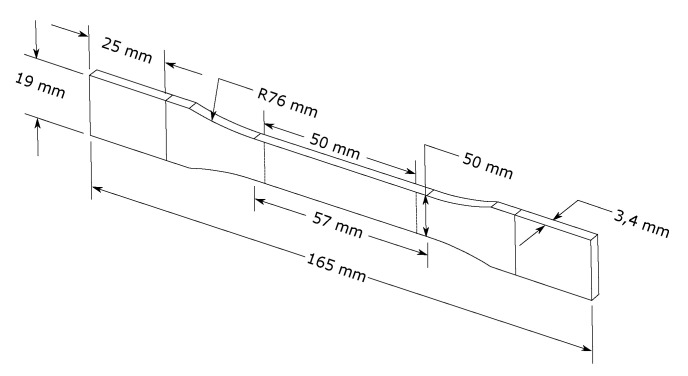
Specimen used for the determination of the S-N curve of the material, in agreement with ASTM D638-14 reference standards.

To correctly estimate the fatigue life of mechanical component, an essential parameter, beside a correct representation of the dynamic model, is the S-N curve of the material or of the component. In order to numerically estimate the fatigue life of the Y-shaped specimen, it was necessary to determine the S-N curve of the adopted material (White pearl PLA manufactured by Ultimaker). For this purpose, 7 dog-bone flat specimens shown in Figure 5 were printed and tested in agreement with ASTM D7791-17 reference standards [60]. The specimens were printed horizontally so as to be consistent with the final printing parameters used for the Y-shaped specimen.

**Figure 6:**
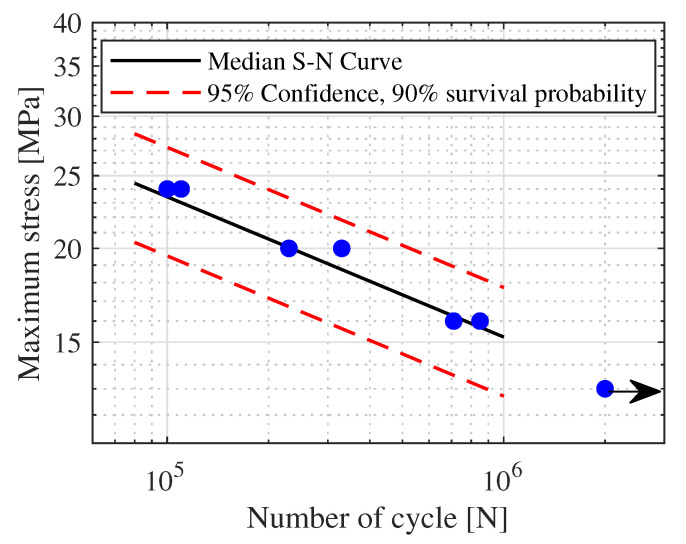
Experimental S-N curve for White Pearl PLA.

All fatigue tests were conducted using a Instron testing machine (Model 1342) performing a constant maximum load stress control tensile-tensile fatigue test. A stress ratio R = 0 was considered. In accordance to the reference standard, a frequency equal to 4 Hz was chosen to identify the fatigue parameters of the material. The fatigue limit was set equal to $2\;\times \;10^{6}$ cycles. Four stress level (24 MPa, 20 Mpa, 16 MPa and 13 MPa) were considered and the relative number of failure cycles were recorded. These tests were used to mainly determine the fatigue limits $\sigma_{lim}^{max}$, the fatigue parameter *k* and *C*. Three survival probabilities (50%, 10%, 90%), were considered. The results of the fatigue test are summarized in the S-N curve shown in Figure 6. The black arrow marks the run-out sample. The run-out represents the test condition for which the sample, subjected to a determine load, withstand for a predefined number of cycle without showing a fatigue crack. In this activity, the number of cycle of the run-out test was set equal to 2$\;\times \;10^{6}$ cycles.

As visible in Figure 6, all the tested specimens fall within the considered probability bands meaning that there is no great variability in the results. From the performed tests, a slope value equal to k=5.3358 and a curve intercept value equal to $C=2.14611\times 10^{12}$ MPa were obtained. About the fatigue limit, the obtained results indicate a value equal to $\sigma_{lim}$ = 11.9 MPa–13.5 MPa–15.3 MPa for 10–50–90% of survival probability respectively.

### 3.4. Numerical Calculation of Fatigue Life Due to Random Vibration

Once the main parameters for the correct description of the dynamic behavior of the structure (damping ratio and elastic modulus) and the S-N curve of the material are known, the fatigue life of the Y-shaped specimen can calculated with the three methods described in Section 2.3.

To this aim, a constant flat Power Spectral Density (PSD), defined between 155 Hz and 230 Hz, thus centered around the fourth natural frequency of the specimen, was used to excite the structure. Three different RMS values were considered: 2.5, 3 and 3.5 g.

**Figure 7:**
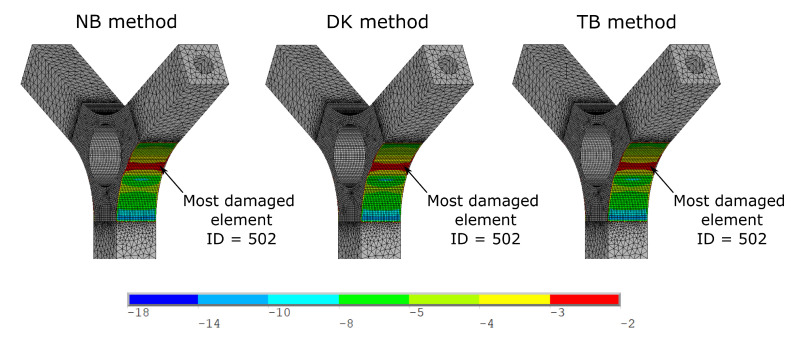
Color map of fatigue damage, in logarithmic scale, computed with the three considered spectral methods for the case of 3.5 g input RMS.

The fatigue life has been computed only for the skin element introduced in Section 3.1, in order to speed up the computational time. For each considered element, the fatigue damage was computed and Figure 7 shows the color map, in logarithmic scale, of the obtained fatigue damage with the three considered spectral methods for the case of 3.5 g input RMS.

As visible in Figure 7, the fatigue damages, computed with the three considered spectral methods, are much similar each other. For all three methods, the most damaged element results to be the element ID = 502. Once the most stressed element is identified, it is possible to compare the results for all the input excitation considered. To this aim, Table 2 shows the obtained fatigue life, in terms of number of cycles, computed with three spectral methods considered for all the input RMS adopted for the element ID = 502.

As visible in Table 2, similar values were obtained with each methods, meaning that all of them can be indiscriminately used for computing the fatigue life of 3D printed structures. Beside the matching between the three methods, an experimental validation of the obtained results is mandatory to check the consistency of the results with the real one. To this aim, in the next section, experimental tests and results are presented.

## 4. Experimental Test for the Validation of Numerical Results

This section describes the procedure of the experimental tests and the obtained outcomes necessary to evaluate the accuracy of the considered spectral methods for the prediction of the fatigue life of a PLA 3D-printed structure.

### 4.1. Specimens and Experimental Setup

In order to evaluate the possibility of estimating the fatigue life of structural components manufactured with PLA through additive manufacturing, a set of 12 specimens were manufactured in thermoplastic material and excited through an electrodynamic shaker until fatigue failure occurs.

**Figure 8:**
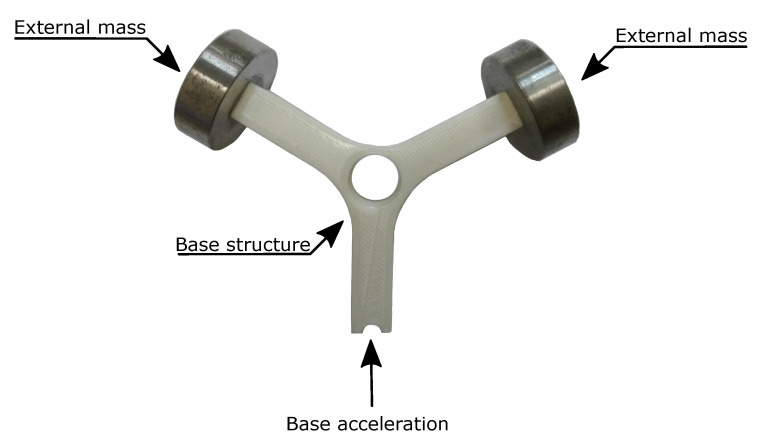
Y-shaped specimen and the adjustable masses used in the experimental activity.

In this activity, a Y-shaped specimen, widely used in random fatigue applications, was used (Figure 8). This is due this structure behaves like a single degree of freedom system and the fatigue crack occurs in a well defined zone of the specimen.

The specimens were printed in White-Pearl PLA (manufactured by Ultimaker) using a Ultimaker S3 printer. The main printing parameters used for the realization of the specimens are: 0.1 mm layer height, 100% infill density (triangular in-fill pattern), 0.1 mm infill layer thickness, 220 °C printing temperature, 60 °C build-plate temperature and at 70 mm/s printing speed.

Since the specimens show several natural frequency, in order to adjust the dynamic behavior of the system (in terms of resonance frequencies) making it suitable for the available instrumentation, two masses of 50 gr each one were screwed to the free ends of the specimen as shown Figure 8.

The two masses were bound to the structure by screws. For this purpose, it was necessary to use iron inserts in order to ensure a stable connection between the parts. The iron inserts were inserted inside two holes of diameter approximately equal to 4.9 mm realized within the structure. An iron solder was used to correctly imbed the iron inserts into the structure. The iron inserts have an internal thread M3 and an external diameter of 5 mm; moreover the outer surface is rough in order to have a connection able to withstand the entire test.

**Figure 9:**
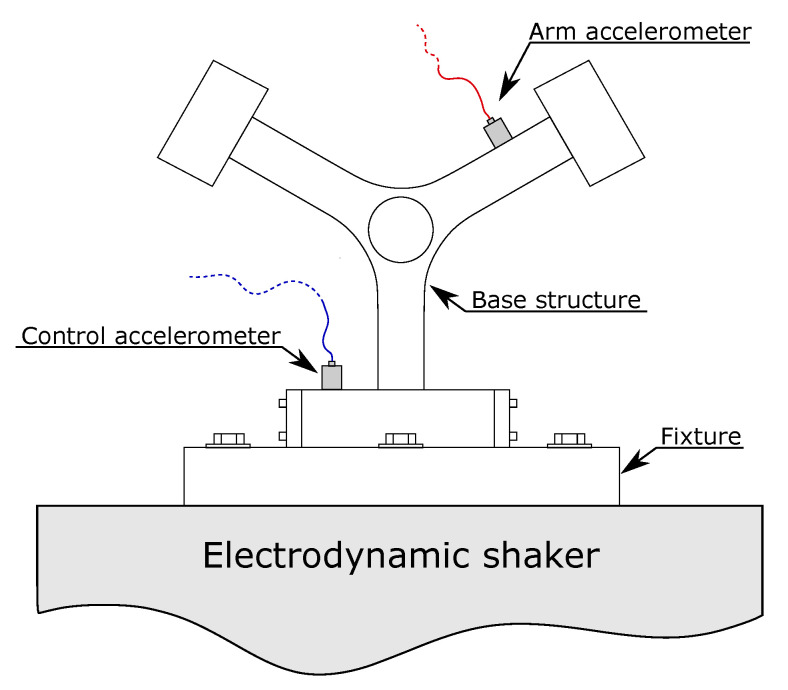
Experimental setup.

The realized Y-shaped specimens were excited with an electrodynamic shaker until a fatigue failure occurs. The experimental setup used in this activity is shown in Figure 9.

As shown in Figure 9, two accelerometers were used. The first, mounted on the fixture, was used to control the excitation profile, while the second accelerometer, installed on the arm, was used to monitor both the dynamic behavior of the system (in terms of frequency response function) and the fatigue life of the component. To this end, the parameter used to highlight component fatigue failure is the shift of the observed natural frequency also called frequency drop. In particular, it was decided to define a fatigue failure of the component when the monitored natural frequency drops down of 5% with respect to its initial value.

To be consistent with what used in the numerical analysis, the same input profile was used in the experimental tests: a constant flat excitation PSD, defined in a frequency range between 155 and 230 Hz, was imposed with three different RMS levels equal to 2.5 g, 3 g and 3.5 g.

### 4.2. Experimental—Numerical Comparison

**Figure 10:**
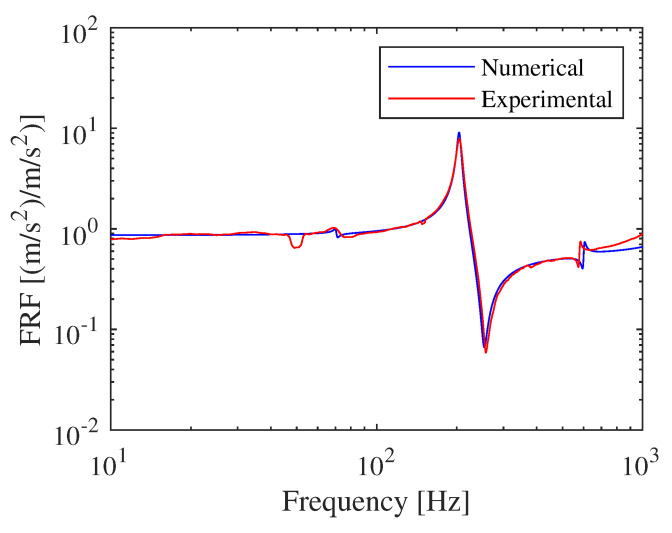
Experimental—numerical comparison of acceleration transfer function.

The first part of the experimental tests was used to compare the dynamic behavior of the realized FE model. To this aim, a comparison between the transfer function between the arm accelerometer and control accelerometer is shown in Figure 10.

As visible in Figure 10, there is a perfect match between the numerical model and the experimental one. The first three vibrating modes of the numerical modal are accurately over-imposed to those experimentally obtained.

Once it has been found a good agreement between the numerical and the experimental model regarding the dynamic behavior, it was possible to compare the fatigue life with those experimentally obtained. As indicated in previous section, the fatigue life of the numerical model has been computed with three of the most used spectral methods (i.e., narrow band method, Dirlik method and Tovo-Benasciutti method). In order to highlight the correspondence between the experimental and numerical obtained fatigue life, Table 3 compares the obtained results.

As visible from Table 3, which compares the experimental and numerical obtained fatigue life, it is possible to note a good agreement between the numerical and experimental results for all the considered excitations. However small variations (even if reasonable) can be noticed between the experimental fatigue life, probably due to the difference in the specimen. The good agreement of the results derived by the three adopted methods can be attributed to dynamic nature of the structure: being a single degree of freedom system, it filters the excitation around the predominant natural frequency (equal to 204 Hz) and thus the stress response tends to be close to a narrow band process. This implies that the correction coefficient foreseen by the Tovo-benasciutti method is close to the unit and thus the NB and TB methods supply very similar results. Further, it has been demonstrated that the Dirlik method is able to provide accurate results for whichever process (both narrow and wide-band) and due to this, also the outcomes of this method are comparable with those derivable by the other two.

To quantify the error committed when the fatigue life is numerically estimated with spectral methods, the absolute percentage error is computed for all the adopted spectral methods and it is shown in Table 4.

**Figure 11:**
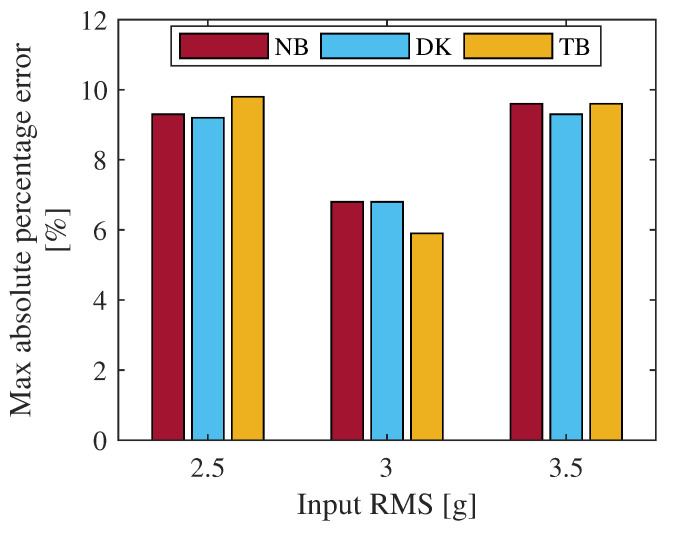
Comparison between the maximum percentage error of fatigue life between the adopted spectral methods for each considered excitation.

As visible from Table 4, the numerical computed fatigue life are in perfect agreement with those experimentally obtained. Indeed, the absolute percentage error shown in Table 4, is always lower than 10%, thus indicating a good correlation between data. To graphically compare the maximum error, Figure 11 shows a comparison, in terms of maximum percentage error of computed fatigue life, between all three adopted spectral methods for fatigue life estimation for each excitation considered in this activity.

**Figure 12:**
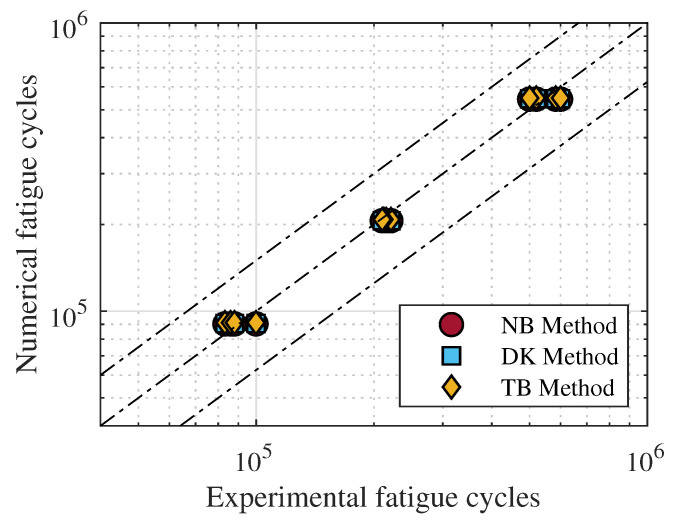
Numerically predicted fatigue life, in terms of number of cycles, compared to those experimentally obtained.

From Figure 11 it is clear as all three spectral methods for fatigue damage estimation supply similar results. The committed errors are comparable to each other. Thus, whichever is the adopted method, the maximum error in fatigue life is lower than 10% at least for the considered cases. To further compare the accuracy of the three adopted spectral methods, a direct comparison between the experimental and numerical obtained fatigue life are depicted in Figure 12.

As clearly emerges from Figure 12, the predicted fatigue life are in line with those experimentally obtained thus certifying a good accuracy of the considered spectral methods for the estimation of fatigue life of 3D printed PLA structures.

## 5. Conclusions and Remarks

Additive manufactured components are, even more frequently, replacing mechanical components fabricated with conventional technique in almost all areas of engineering and for this, they are subjected to dynamic loads, even random.

For this reason, the possibility of using classical spectral methods (despite developed for metal alloys) to determine the fatigue life of additive manufactured mechanical components realized in PLA was evaluated in this activity. To this aim, a Y-shaped specimen, classically used in random fatigue applications, was adopted and the fatigue life was predicted using three of the most commonly used spectral methods (Narrow band, Dirlik, Tovo-Benasciutti). To correctly estimate the dynamic behavior and the fatigue life, the dynamic parameters of the material were firstly determined by DMA test and subsequently the S-N curve was computed according with ASTM D7791-17 reference standards.

Twelve specimens were experimentally tested through a electrodynamic shaker until fatigue failure occurs, for three different RMS values of the excitation. The obtained results show an excellent agreement between the experimental and numerical results. For all the excitation profiles considered, the investigated spectral methods grant a good accuracy of the result in terms of fatigue life. The maximum committed percentage error is always lower than 10%, certifying the reliability of the considered methods to predict the fatigue life of PLA 3D printed structures.

## Figures and Tables

**Table 1:** Average results of Elastic modulus, loss factor and damping ratio obtained through DMA.

	Elastic Modulus $E_{b}$	Loss Factor ι	Damping Ratio ξ
	[MPa]	[-]	[-]
Horizontal printing	2805	0.0158	0.008

**Table 2:** Numerical fatigue life obtained with three spectral methods considered in this activity for the element ID = 502.

Test ID	Input RMS	NB	DK	TB
[-]	[g]	[Cycles]	[Cycles]	[Cycles]
1	3.0	$2.06\;\times \;10^{5}$	$2.06\;\times \;10^{5}$	$2.08\;\times \;10^{5}$
2	3.0	$2.06\;\times \;10^{5}$	$2.06\;\times \;10^{5}$	$2.08\;\times \;10^{5}$
3	3.0	$2.06\;\times \;10^{5}$	$2.06\;\times \;10^{5}$	$2.08\;\times \;10^{5}$
4	3.0	$2.06\;\times \;10^{5}$	$2.06\;\times \;10^{5}$	$2.08\;\times \;10^{5}$
5	3.5	$9.03\;\times \;10^{4}$	$9.06\;\times \;10^{4}$	$9.12\;\times \;10^{4}$
6	3.5	$9.03\;\times \;10^{4}$	$9.06\;\times \;10^{4}$	$9.12\;\times \;10^{4}$
7	3.5	$9.03\;\times \;10^{4}$	$9.06\;\times \;10^{4}$	$9.12\;\times \;10^{4}$
8	3.5	$9.03\;\times \;10^{4}$	$9.06\;\times \;10^{4}$	$9.12\;\times \;10^{4}$
9	2.5	$5.44\;\times \;10^{5}$	$5.46\;\times \;10^{5}$	$5.49\;\times \;10^{5}$
10	2.5	$5.44\;\times \;10^{5}$	$5.46\;\times \;10^{5}$	$5.49\;\times \;10^{5}$
11	2.5	$5.44\;\times \;10^{5}$	$5.46\;\times \;10^{5}$	$5.49\;\times \;10^{5}$
12	2.5	$5.44\;\times \;10^{5}$	$5.46\;\times \;10^{5}$	$5.49\;\times \;10^{5}$

**Table 3:** Comparison between the experimental fatigue life, in terms of number of cycle, and those numerical obtained with the three used spectral methods.

Test ID	Input RMS	Experimental	NB	DK	TB
[-]	[g]	[Cycles]	[Cycles]	[Cycles]	[Cycles]
1	3.0	$2.15\;\times \;10^{5}$	$2.06\;\times \;10^{5}$	$2.06\;\times \;10^{5}$	$2.08\;\times \;10^{5}$
2	3.0	$2.21\;\times \;10^{5}$	$2.06\;\times \;10^{5}$	$2.06\;\times \;10^{5}$	$2.08\;\times \;10^{5}$
3	3.0	$2.12\;\times \;10^{5}$	$2.06\;\times \;10^{5}$	$2.06\;\times \;10^{5}$	$2.08\;\times \;10^{5}$
4	3.0	$2.10\;\times \;10^{5}$	$2.06\;\times \;10^{5}$	$2.06\;\times \;10^{5}$	$2.08\;\times \;10^{5}$
5	3.5	$9.99\;\times \;10^{4}$	$9.03\;\times \;10^{4}$	$9.06\;\times \;10^{4}$	$9.12\;\times \;10^{4}$
6	3.5	$8.32\;\times \;10^{4}$	$9.03\;\times \;10^{4}$	$9.06\;\times \;10^{4}$	$9.12\;\times \;10^{4}$
7	3.5	$8.60\;\times \;10^{4}$	$9.03\;\times \;10^{4}$	$9.06\;\times \;10^{4}$	$9.12\;\times \;10^{4}$
8	3.5	$8.80\;\times \;10^{4}$	$9.03\;\times \;10^{4}$	$9.06\;\times \;10^{4}$	$9.12\;\times \;10^{4}$
9	2.5	$5.83\;\times \;10^{5}$	$5.44\;\times \;10^{5}$	$5.46\;\times \;10^{5}$	$5.49\;\times \;10^{5}$
10	2.5	$6.00\;\times \;10^{5}$	$5.44\;\times \;10^{5}$	$5.46\;\times \;10^{5}$	$5.49\;\times \;10^{5}$
11	2.5	$5.20\;\times \;10^{5}$	$5.44\;\times \;10^{5}$	$5.46\;\times \;10^{5}$	$5.49\;\times \;10^{5}$
12	2.5	$5.00\;\times \;10^{5}$	$5.44\;\times \;10^{5}$	$5.46\;\times \;10^{5}$	$5.49\;\times \;10^{5}$

**Table 4:** Absolute percentage error of the computed fatigue life with the adopted spectral methods.

Test ID	Input RMS	NB Error	DK Error	TB Error
[-]	[g]	[%]	[%]	[%]
1	3.0	4.2	4.2	3.3
2	3.0	6.8	6.8	5.9
3	3.0	2.8	2.8	1.9
4	3.0	1.9	1.9	1.0
5	3.5	9.6	9.3	8.7
6	3.5	8.5	8.9	9.6
7	3.5	5.0	5.3	6.0
8	3.5	2.6	3.0	3.6
9	2.5	6.7	6.3	5.8
10	2.5	9.3	9.0	8.5
11	2.5	4.6	5.0	5.6
12	2.5	8.8	9.2	9.8

## Data Availability

Not applicable.
